# Implicit Attitudes About Agricultural and Aquatic Products From Fukushima Depend on Where Consumers Reside

**DOI:** 10.3389/fpsyg.2019.00515

**Published:** 2019-03-13

**Authors:** Otgonchimeg Tsegmed, Daiki Taoka, Jiang Qi, Atsunori Ariga

**Affiliations:** ^1^ Graduate School of Integrated Arts and Sciences, Hiroshima University, Higashihiroshima, Japan; ^2^ Kyoto University, Kyoto, Japan

**Keywords:** Fukushima nuclear disaster, purchase hesitation, implicit attitude, perceived vulnerability to disease, behavioral immune system

## Abstract

Japanese consumers are still hesitant to purchase products from Fukushima, although 7 years have passed since the Fukushima nuclear disaster, and these products are officially considered safe. In this study, we examined whether Japanese consumers have negative implicit attitudes toward agricultural and aquatic products from the Fukushima region and whether these attitudes are independent of their explicit attitudes. Japanese students completed an implicit association test and a questionnaire to assess their implicit and explicit attitudes toward products from Fukushima relative to another region. The results of two experiments reliably demonstrated that the public has negative implicit attitudes toward Fukushima products, whereas their explicit attitudes are consistently positive. These observations predominantly held for participants living close to Fukushima (Tokyo) as opposed to participants living far away (Hiroshima): Experiment 1 (*n* = 40). Furthermore, individual differences in aversion to germs contributed to the implicit attitudes; the implicit negative attitudes were attenuated among the participants with a relatively low aversion to germs: Experiment 2 (*n* = 60). These results suggest that the implicit attitudes associated with the behavioral immune system, which is conceptualized as a suite of psychological mechanisms designed to proactively resist pathogenic threats, may underlie the hesitation to purchase products from Fukushima.

## Introduction

Although more than several years have passed since the Tohoku Earthquake and the Fukushima Daiichi nuclear power plant disaster, which occurred in 2011, Japanese consumers are still hesitant to purchase agricultural and aquatic products from Fukushima (Hangui, 2014; Fukushima Prefecture, 2016; Consumers Affairs Agency, 2017). It may have been advisable to refrain from consuming Fukushima products immediately after the disaster because of the widespread and unreliable, reputational damaging information regarding radioactive contamination. However, these products are now officially safe to buy (Fukushima Prefecture, 2013). Hence, consumers no longer have any grounds for avoiding them. Nevertheless, this hesitancy, which is a response to the social stigma against Fukushima products, persists; social stigma is defined as the disapproval of someone or something based on perceivable social characteristics used to differentiate it from others (Crocker and Major, 1989). This is causing serious economic damage (i.e., a collapse in the price, Central Union of Agricultural Co-operatives, 2011; Fukushima Minyu Shimbun Sha, 2012; Ichinose, 2012). For example, the market prices of beef, peaches, and rice, which are specialties from the Fukushima region, have continuously decreased since 2011 (e.g., by 9.3, 4.9, and 23.3% in 2017) with respect to the national average because of the disaster (Reconstruction Agency, 2018).

Such hesitancy in purchasing products from Fukushima can be interpreted in the context of error management theory (Haselton and Buss, 2000). According to this theory, an individual makes two possible errors (type I being false-positive and type II being false-negative errors) when making a decision in an uncertain scenario. Essentially, consumers tend to be afraid of making a type II error judgment, in which they mistake products that are dangerous as being safe. Instead, type I error judgments, where safe products are mistaken as dangerous, are more likely. In short, this hesitancy is caused by consumers’ vigilance about products from Fukushima.

To prevent reputational damage to products from Fukushima and correct consumers’ overcautious attitudes toward them, local and national governments have repeatedly released information regarding their safety, evidenced by screening for radioactive contamination (Ministry of Health, Labour and Welfare, 2018). Due to such attempts, the latest survey research demonstrates that the number of consumers who care about the production area has drastically decreased and that nearly 80% of consumers have no concerns about the safety of Fukushima products (Ministry of Agriculture, Forestry, and Fisheries, 2018). Furthermore, consumers do not currently have “explicitly” negative attitudes toward products from Fukushima, at least on paper (Miura et al., 2016; Kudo and Nagaya, 2017). If this is so, then why do they still hesitate to purchase these products? It appears that another factor, which we consider to be their “implicit” attitudes, underlies this hesitation. It has been suggested that explicit and implicit attitudes differ from one another, particularly with respect to social stigma (Wilson et al., 2000). Thus, we hypothesized that implicit and explicit attitudes toward products from Fukushima are dissociated from one another and that, rather than explicit attitudes, negative implicit attitudes underlie the hesitancy regarding the purchase of Fukushima products.

However, before we can resolve this major hypothesis, it is necessary to validate the basic aspects of our hypothesis, namely: whether consumers indeed have negative implicit attitudes toward agricultural and aquatic products from the Fukushima region and whether these attitudes are independent of their explicit attitudes. We used the implicit association test (IAT), which is a well-known method for measuring implicit attitudes related to a target attribute relative to another (Greenwald et al., 1998). In this study, the participants completed both an IAT and a questionnaire to assess their implicit and explicit attitudes toward products from Fukushima relative to products from another region.

Note that recent research based on the IAT has already reported that Japanese people show somewhat positive, not negative, implicit attitudes toward products from Fukushima (Kudo and Nagaya, 2017). However, we believe that the interpretation of the data from that is limited for the following two reasons. First, the study focused on the effects of a persuasive message on consumers’ attitudes, and thus, the implicit attitudes were only measured after manipulating the participants with this message, and the questionnaire measures explicit attitudes. Thus, it was likely that the participants’ implicit attitudes had been biased by the exposure to the preceding message and/or the questionnaire. Second, the authors did not provide any information regarding where the participants lived. Because the importance of (or the amount of exposure to) the Fukushima brand increases as the consumers’ physical distance from the area where the disaster occurred decreases (Miura et al., 2016), it is plausible that consumers’ attitudes toward Fukushima depend on where they live. It has not yet been determined whether consumers’ implicit attitudes are modulated by where they live. In this study, excluding the potential confounding factors mentioned, we investigated whether explicit and implicit attitudes vary as a function of location. This is obviously an important factor to be taken into account when marketing products from Fukushima.

## Experiment 1

In the first experiment, we investigated (1) whether consumers have negative implicit attitudes toward products from the Fukushima region, (2) whether these are independent of their explicit attitudes, and (3) whether a consumer’s attitude is modulated by where they live. First, the participants completed the IAT. This provided a measure of their implicit attitudes. Then, they answered a questionnaire that measured their explicit attitudes. We recruited participants from two geographically distant areas (Hiroshima and Tokyo, which are 811 and 239 km away from Fukushima as the crow flies). Then, we compared the participants’ attitudes.

### Method

*Ethics Statement* All of the experiments carried out in this study were reviewed and approved by the Institutional Review Boards of Hiroshima University (Hiroshima) and Rissho University (Tokyo), Japan. Written informed consent was obtained from all participants before and after the experiment.

*Participants* We recruited 20 Japanese participants (12 female, mean age = 20.60 years, *SD* = 1.43 years) from Hiroshima and 20 Japanese participants (10 female, mean age = 20.00 years, *SD* = 0.45 years) from Tokyo with the aim of investigating human cognition. We ran the experiments in Hiroshima and Tokyo in parallel, and they took place between November 2017 and April 2018. The participants were blinded to the purpose of the study.

*Stimuli* We used 12 full-color images as stimuli: four of aquatic products and eight of agricultural products (four flowers and four rice). All images were made in our laboratory. Two aquatic products, two flowers, and two rice images were randomly selected and labeled with the kanji for “Fukushima product.” We labeled the products because the images themselves do not convey information regarding where they were produced. The other six images were labeled with the kanji for “Saga product.” The word label was white and was placed below the image. In addition, we used five positive word labels and five negative word labels (10 words total) as stimuli, based on Ishii and Numazaki (2009). These labels were written in white with kanji or hiragana scripts. Each stimulus was presented twice within a block. The visual angle of each image subtended 11 × 11° and each character subtended approximately 1.5 × 1.5°. The stimuli were presented at the center of the screen on a black background. The participants’ viewing distance was about 57 cm.

We selected Saga prefecture, which is 1,048 km away from Fukushima prefecture, as the reference region. This is because Saga was ranked at a very similar position to Fukushima in the Japanese prefecture attractiveness rankings (Brand Research Institute, 2016) and that, like Fukushima, Saga specializes in agricultural and aquatic products. The participants of our pilot study (*n* = 8 in Hiroshima, *n* = 8 in Tokyo) also evaluated Saga neutrally; participants in Hiroshima rated 3.00 on average (*SD* = 0.50) and those in Tokyo rated 2.88 on average (*SD* = 0.60) to the question, “How much do you favor Saga prefecture relative to Fukushima prefecture? (1 = not favorable at all, 5 = very favorable).”

*Procedure* The participants performed the IAT task individually. The IAT was conducted in a laboratory under dimmed lighting conditions. We followed the IAT procedure developed by Greenwald et al. (1998), which consists of seven blocks. In each of the blocks, the participants were required to categorize the presented target stimulus by pressing either the left (F) key or the right (J) key on the keyboard using their two index fingers as quickly and accurately as possible. The target stimulus remained on the screen until participants provided a response. A red cross appeared in the center of the screen when participants pressed the wrong key.

In Block 1, which consisted of 24 training trials, the participants were trained to discriminate between products labeled from Fukushima and products from Saga: the left key indicated a Fukushima product and the right key indicated a product from Saga. In Block 2 (20 training trials), the participants were trained to determine whether the meaning of the word label was positive or negative: pressing the left key for positive words and the right key for negative words. In Block 3, which consisted of 22 practice trials, and Block 4, which included 44 test trials, we combined Fukushima/Saga and positive/negative discriminations: pressing the left key for Fukushima or positive words and the right key for Saga or negative words. In the following blocks, the participants learned the opposite category-key mapping to that of Blocks 1, 3, and 4. In Block 5 (24 training trials), they were trained to indicate whether the product image was labeled as being from Saga or Fukushima: pressing the left key for Saga and the right key for Fukushima. Then, in Block 6 (22 practice trials) and Block 7 (44 test trials), the participants pressed the left key when the presented stimulus was labeled as being from Saga or with a positive word, whereas they pressed the right key when the stimulus was labeled with Fukushima or a negative word. The order of the combinations was counterbalanced between the participants; blocks 1, 3, and 4 were switched with blocks 5, 6, and 7 for half of the participants.

Before each block, the participants were fully informed of the next task. Furthermore, we reminded the participants of the category-key mapping for each block as it came, and cue words were presented on the left-top or right-top of the screen for each trial. After completing the IAT, the participants were requested to answer a questionnaire on paper, consisting of two items that assessed the absolute likeability of Fukushima and Saga on an 11-point Likert scale and one item that assessed their relative likeabilities on a 7-point Likert scale: (1) How much do you like products from Fukushima? (−5 = “I do not like Fukushima products at all,” +5 = “I like Fukushima products very much”). (2) How much do you like products from Saga? (−5 = “I do not like Saga products at all,” +5 = “I like Saga products very much”). (3) Which production area do you prefer? (−3 = “I strongly prefer Saga to Fukushima,” +3 = “I strongly prefer Fukushima to Saga”). The neutral point was 0 for all three questions.

### Data Analyses

As traditional null hypothesis significance tests do not allow for evidence in favor of the null hypothesis (Wagenmakers, 2007; Cumming, 2013), we used default Bayesian tests (Rouder and Morey, 2012) to determine whether the production area had any effect or whether attitudes leaned to one side (positive or negative) or not (neutral). We treated the Bayes factors as measures of evidence for or against the effects of interest. Briefly, a Bayes factor (*B_10_*) indicates the ratio of the likelihood that the data obtained favor a statistical model, including the effects of interest, to the likelihood that they favor a model that excludes these effects. We use the terminology from Jeffreys (1961) and Wagenmakers et al. (2011) to denote the magnitude of the effects. A *B_10_* value >1 provides evidence for a statistical effect, whereas a *B_10_* value <1 provides evidence for the null hypothesis.

As an index of the positivity/negativity of the implicit attitudes toward Fukushima products relative to Saga products, we calculated the *D* score for each participant based on the reaction times (RTs) during the IAT (Greenwald et al., 2003; Lane et al., 2007, for more details). First, we excluded incorrect trials and trials with RTs < 300 ms and > 10,000 ms from the following analyses; consequently, 4.08% of the trials were excluded on average. Second, for each pair of practice (blocks 3 and 6) and test blocks (blocks 4 and 7), we subtracted the mean RTs from the Fukushima-positive/Saga-negative combined task from the mean RTs from the Fukushima-negative/Saga-positive combined task. Third, each difference was divided by the overall standard deviation of the RTs for that pair of blocks. Finally, we calculated the equal weight average from the two ratios (mean differences/standard deviation) to obtain the *D* score. That is, a *D* score < 0 means that the Fukushima-negative/Saga-positive association is stronger than the Fukushima-positive/Saga-negative association, whereas a *D* score > 0 means the reverse.

To compute internal consistency, we first separately subtracted each trial’s RT in the Fukushima-negative/Saga-positive combination block from the RT of the corresponding trial of the Fukushima-positive/Saga-negative block. We then computed Cronbach’s alpha (Cronbach and Meehl, 1955) of these different scores, following Bosson et al. (2000). Cronbach’s alpha is the most routinely used method to estimate the reliability of internal consistency (Parsons et al., 2018). Alpha indicates the internal consistency in the tendency to associate Fukushima-related stimuli with a negative word (or impression) relative to Saga-related stimuli.

### Results

**Figure 1:**
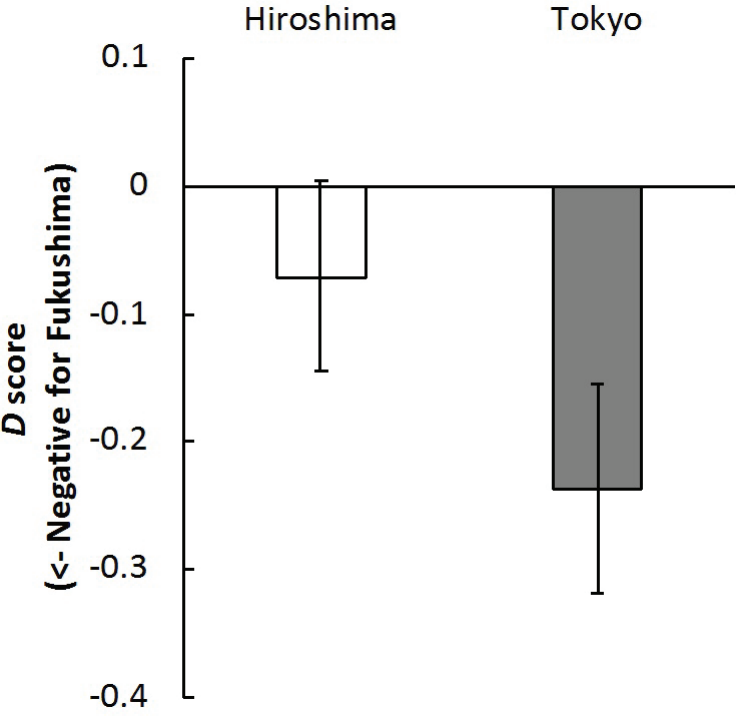
Mean *D* scores in Hiroshima and Tokyo in Experiment 1. Error bars indicate standard error of the mean.

*Implicit Attitudes* The mean *D* scores of participants from Hiroshima and Tokyo were both less than 0, as shown in Figure 1. According to the results of a Bayesian one sample *t* test, there was moderate evidence that the score favored the null hypothesis in Hiroshima (*B_10_* = 1/3.03, Cohen’s *d* = 0.20), whereas there was moderate evidence that the score was less than 0 in Tokyo (*B_10_* = 4.66, Cohen’s *d* = 0.62). The Cronbach’s alpha values were 0.91 for the IAT results of Hiroshima and 0.57 for Tokyo.

**Figure 2:**
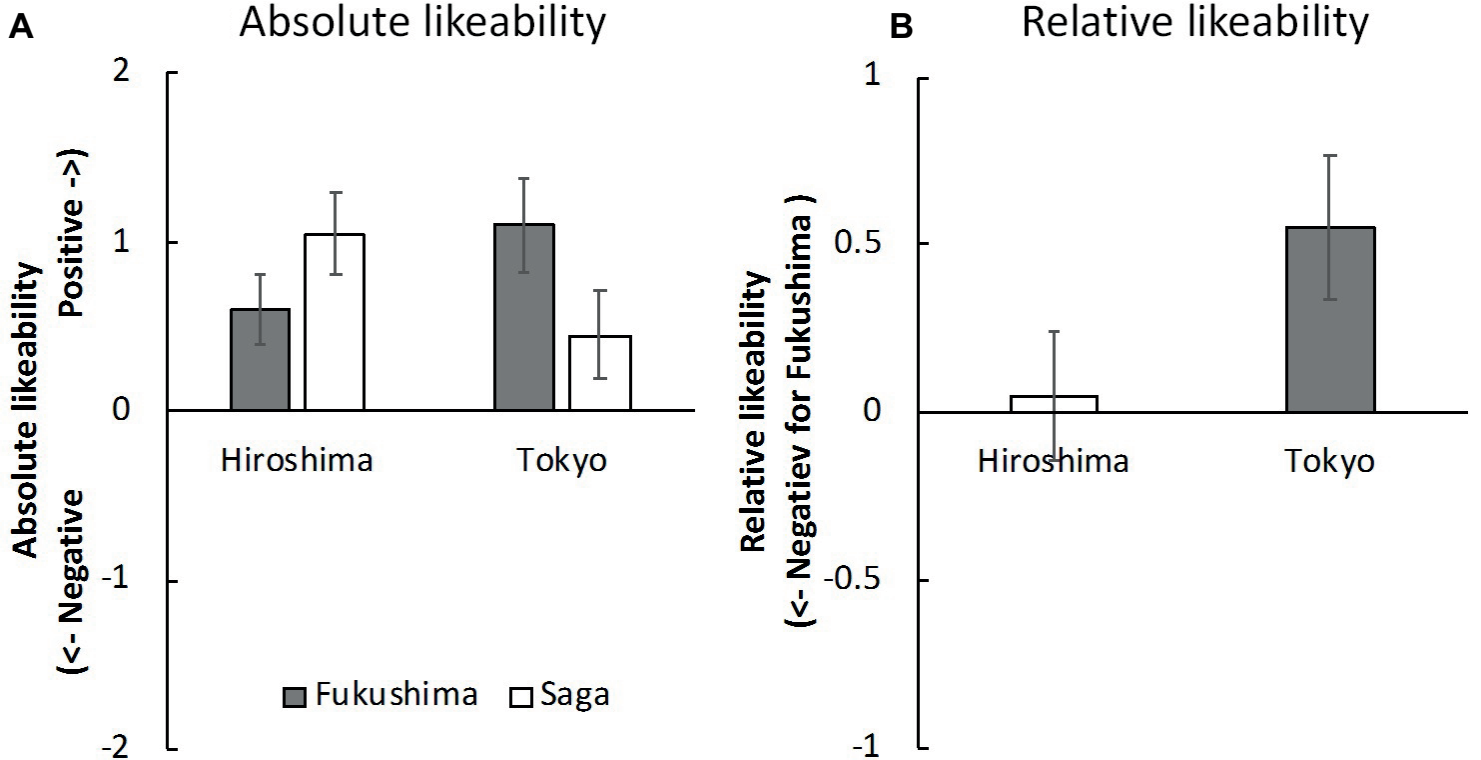
Mean rating scores of **(A)** absolute likeability of Fukushima and Saga in Hiroshima and Tokyo and **(B)** relative likeability of Fukushima to Saga in Hiroshima and Tokyo. The neutral point is zero. Error bars indicate standard error of the mean.

*Explicit Attitudes* We calculated the mean rating scores of absolute likeability (Figure 2A) and relative likeability (Figure 2B). For the participants from Hiroshima, Bayesian one sample *t* tests demonstrated moderate and strong evidence that the rating scores of absolute likeability were greater than 0 for products from Fukushima (*B_10_* = 5.05, Cohen’s *d* = 0.63) and Saga (*B_10_* = 79.55, Cohen’s *d* = 0.95). There was strong evidence that the participants from Tokyo rated the absolute likeability of Fukushima with a score greater than 0 (*B_10_* = 39.96, Cohen’s *d* = 0.87), but anecdotal evidence that the absolute likeability of products from Saga favored the null hypothesis (*B_10_* = 1/1.29, Cohen’s *d* = 0.38). In the case of the relative likeability, Hiroshima residents held moderately neutral attitudes (*B_10_* = 1/4.34, Cohen’s *d* = 0.05), whereas Tokyo residents anecdotally preferred Fukushima to Saga (*B_10_* = 2.53, Cohen’s *d* = 0.55).

### Discussion

In Hiroshima, the Fukushima-negative/Saga-positive implicit association was equivalent to the Fukushima-positive/Saga-negative implicit association, suggesting that Hiroshima residents have neutral (or Saga-level) implicit attitudes toward Fukushima products; internal consistency was acceptable based on Cronbach’s alpha. They held explicitly positive attitudes toward products from both regions. These results are consistent with those of previous work (Miura et al., 2016; Kudo and Nagaya, 2017) that found that Japanese consumers in general do not currently have negative implicit or explicit attitudes toward products from Fukushima.

On the other hand, the Fukushima-negative/Saga-positive implicit association was stronger than the Fukushima-positive/Saga-negative implicit association in the case of Tokyo residents; internal consistency was slightly low, which may have been due to the small sample size (but see acceptable evidence observed in Experiment 2 below). This suggests that they have negative implicit attitudes toward products from Fukushima relative to those from Saga. Interestingly, their explicit attitudes were inconsistent with their implicit attitudes, which were consistently positive toward Fukushima. This discrepancy is in line with the previous suggestion (Wilson et al., 2000) that explicit and implicit attitudes can differ from one another, particularly with respect to social stigma.

Although we presented identical stimuli and tasks and used identical recruiting of participants in Hiroshima and Tokyo, the results of the IAT were different. This demonstrates that our current findings cannot be attributed to the physical characteristics of the stimulus images. Therefore, the results of Experiment 1 suggest that consumers have relatively negative implicit attitudes toward products from Fukushima, although their explicit attitudes are positive. This divergence was predominantly observed in the region near to Fukushima (i.e., Tokyo). These findings support our hypothesis that it is implicit negative attitudes rather than explicit negative attitudes that underlie the hesitancy to purchase products from Fukushima.

We built on the findings from Experiment 1 in the next experiment, in which we explored the individual differences between the negative implicit attitudes toward products from Fukushima held by Tokyo residents. We focused on the *perceived vulnerability to disease* (PVD, Duncan et al., 2009), which is defined as an individual’s beliefs about their subjective vulnerability to the transmission of infectious diseases (*perceived infectability*) and an individual’s tendency to experience emotional discomfort when exposed to potential disease transmission (*germ aversion*). Because negative implicit and explicit social attitudes are associated with individual differences between chronic and temporary concerns about disease transmission (Faulkner et al., 2004; Schaller and Duncan, 2007; Duncan et al., 2009; Duncan and Schaller, 2009; Huang et al., 2011; Murray et al., 2013), it is possible that consumers’ implicit attitudes toward products from Fukushima, or radioactive contamination in Fukushima, would vary with their PVD. Although the effects of radiation are not infectious, they are parallel to pathogenic threats in terms of being invisible. Previous research on evolutionary psychology proposes an adaptive strategy, which is conceptualized as the *behavioral immune system*. This allows us to detect and avoid objects perceived as a threat to health (Kurzban and Leary, 2001; Schaller and Duncan, 2007; Schaller and Park, 2011). Within this context, we predicted that the implicit attitudes toward Fukushima products may depend on the individual differences between the participants’ aversive affective responses to an invisible threat, particularly germ aversion, in terms of their PVD.

## Experiment 2

### Method

*Participants* We recruited 60 Japanese participants (44 female, mean age = 19.88 years, *SD* = 6.84 years) from Tokyo with the aim of investigating human cognition. The experiment was run between April 2018 and July 2018. The participants were blinded to the purpose of this study.

*Stimuli and Procedure* These were almost the same as those used in Experiment 1, except that the participants completed the Japanese version of the PVD questionnaire (Fukukawa et al., 2014) at the end of the experiment. This was composed of two subscales used to assess perceived infectability and germ aversion, as in Duncan et al. (2009).

### Results

*PVD* The perceived infectability ranged from 1.00 to 6.14 (*M* = 3.75, *SD* = 1.29), and germ aversion ranged from 2.13 to 6.38 (*M* = 4.14, *SD* = 0.98).

*Implicit Attitudes and PVD* We first calculated the *D* score, as in Experiment 1, which demonstrated extreme evidence that the score was less than 0 (*M* = −0.28, *SE* = 0.04, *B_10_* = 391.99 × 10^3^, Cohen’s *d* = 0.82); Cronbach’s alpha was 0.92. The correlation between the *D* score and perceived infectability demonstrated moderate evidence for the null hypothesis (*B_10_* = 1/5.46, *r* = −0.07). The correlation between the *D* score and the germ aversion score also demonstrated moderate evidence for the null hypothesis (*B_10_* = 1/3.64, *r* = −0.14), though it was negative.

**Figure 3:**
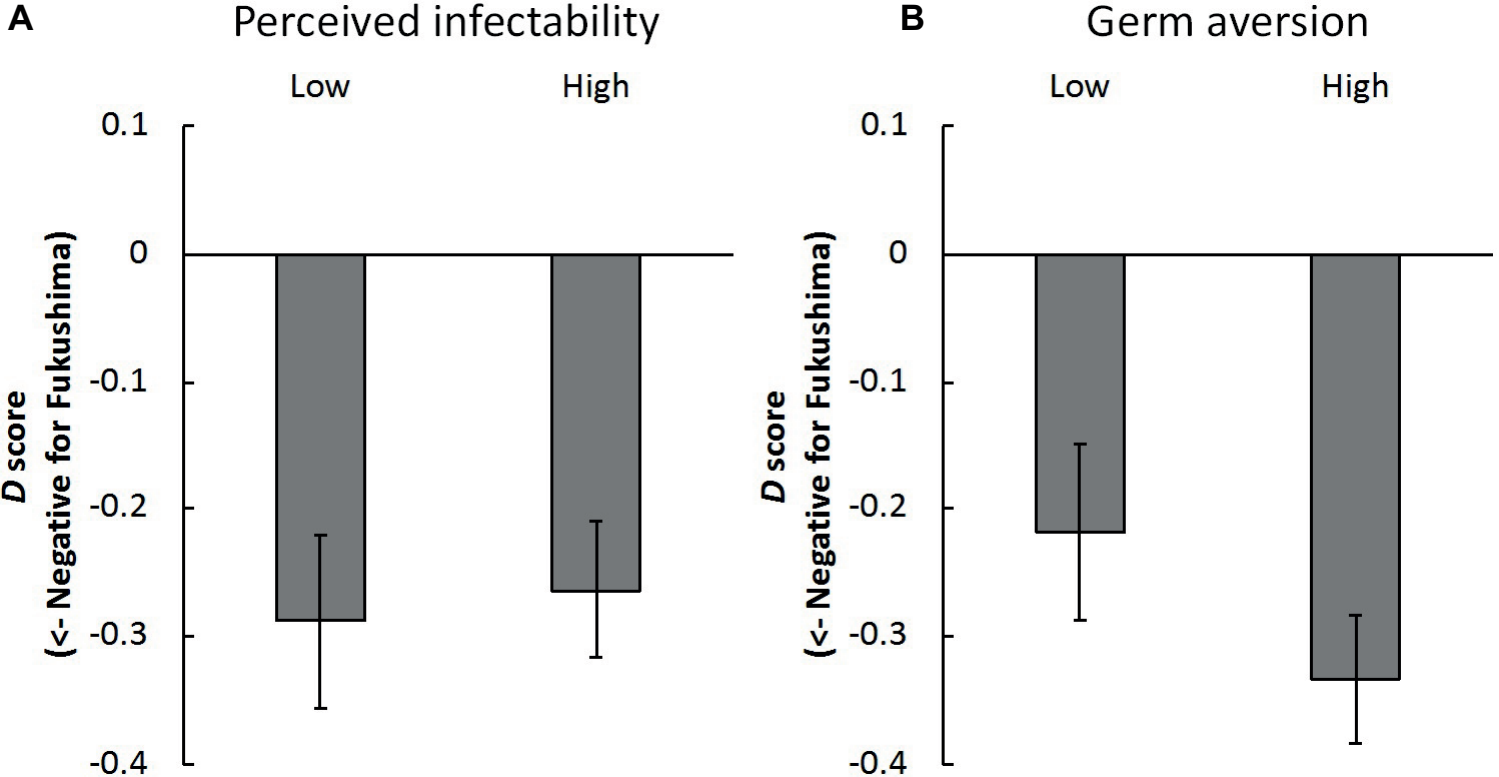
Mean *D* scores among Tokyo residents of **(A)** low/high perceived infectability and **(B)** low/high germ aversion in Experiment 2. Error bars indicate standard error of the mean.

To assess the influence of perceived infectability from a different perspective, we divided the participants into a low group and a high group (with average scores of 2.70 vs. 4.80, *n* = 30 in each group, see Figure 3A). We conducted Bayesian one sample *t* tests, which revealed strong evidence that the *D* scores were less than 0, independently of perceived infectability (*B_10_* = 107.18, Cohen’s *d* = 0.75 for low-score group; *B_10_* = 808.47, Cohen’s *d* = 0.90 for high-score group). In terms of the germ aversion score (3.36 vs. 4.91 in average, *n* = 30 in each group, as shown in Figure 3B), the high-score participants demonstrated extremely negative implicit attitudes toward products from Fukushima (*B_10_* = 539.93 × 10^2^, Cohen’s *d* = 1.20). There was only moderate evidence among the low-score participants (*B_10_* = 9.72, Cohen’s *d* = 0.57).

*Explicit Attitudes and PVD* We calculated the absolute and relative likeabilities, which demonstrated extreme evidence that all of the scores were above 0 (*M* = 2.33, *SE* = 0.18, *B_10_* = 247.20 × 10^13^, Cohen’s *d* = 1.63 for the absolute likeability of Fukushima; *M* = 1.27, *SE* = 0.20, *B_10_* = 424.55 × 10^3^, Cohen’s *d* = 0.82 for the absolute likeability of Saga; *M* = 0.60, *SE* = 0.13, *B_10_* = 830.98, Cohen’s *d* = 0.59 for the relative likeability of Fukushima to Saga). The correlation between the explicit attitudes and perceived infectability demonstrated evidence for the null hypothesis (*B_10_* < 1/1.16, *r* = −0.24 to 0.12). The correlation between the explicit attitudes and germ aversion also demonstrated evidence for the null hypothesis (*B_10_* < 1/3.33, *r* = −0.15 to −0.05).

**Figure 4:**
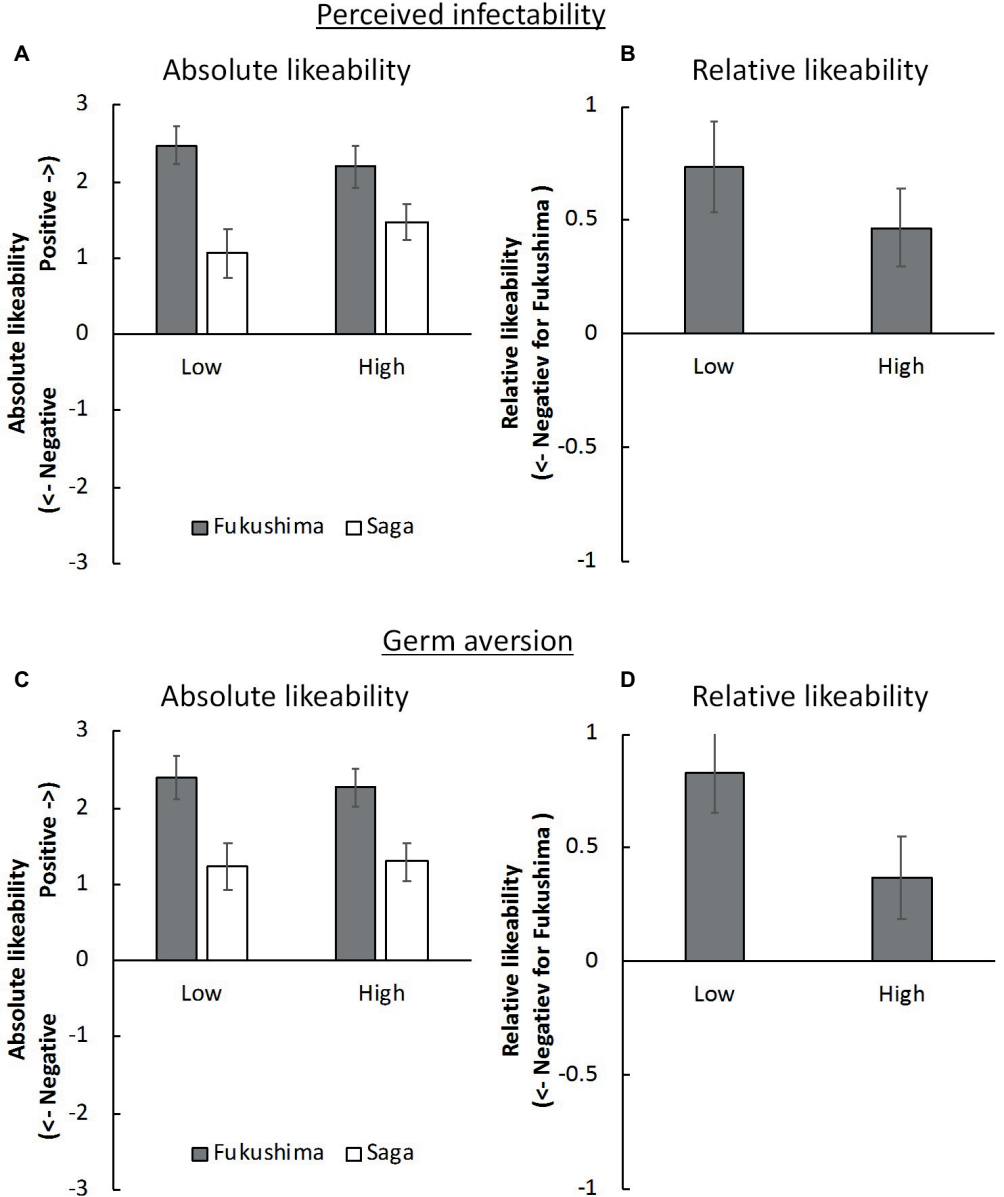
Mean rating scores of **(A)** absolute likeability of Fukushima and Saga and **(B)** relative likeability in each low/high perceived infectability group and of **(C)** absolute likeability and **(D)** relative likeability in each low/high germ aversion group. Error bars indicate standard error of the mean.

We calculated the mean rating scores, in terms of perceived infectability and germ aversion, for the absolute and relative likeability for both the low group and the high group. The results of our Bayesian one sample *t* tests demonstrated strong evidence that the absolute likeability rating scores were greater than 0 (Figure 4A). These were less influenced by the perceived infectability (*B_10_* = 133.30 × 10^6^, Cohen’s *d* = 1.81 for Fukushima in the low-score group; *B_10_* = 15.12, Cohen’s *d* = 0.60 for Saga in low-score group; *B_10_* = 138.40 × 10^4^, Cohen’s *d* = 1.45 for Fukushima in the high-score group; *B_10_* = 232.34 × 10^2^, Cohen’s *d* = 1.14 for Saga in the high-score group). We have strong evidence that the relative likeability rating was greater than 0 among the low-perceived-infectability participants (Figure 4B, *B_10_* = 37.93, Cohen’s *d* = 0.67), whereas this evidence was moderate among the high-perceived-infectability participants (*B_10_* = 4.24, Cohen’s *d* = 0.49).

The absolute likeability rating scores were much greater than 0 (Figure 4C), being less influenced by germ aversion (*B_10_* = 708.40 × 10^4^, Cohen’s *d* = 1.57 for Fukushima in the low-score group; *B_10_* = 64.21, Cohen’s *d* = 0.71 for Saga in the low-score group; *B_10_* = 208.80 × 10^5^, Cohen’s *d* = 1.66 for Fukushima in the high-score group; *B_10_* = 151.18 × 10, Cohen’s *d* = 0.95 for Saga in the high-score group). We have strong evidence that the relative likeability rating was greater than 0 among the low germ aversion participants (Figure 4D, *B_10_* = 355.65, Cohen’s *d* = 0.84), whereas our evidence was anecdotal in the case of the high germ aversion participants (*B_10_* = 1.12, Cohen’s *d* = 0.36).

*Correlation Between Implicit and Explicit Attitudes* Because, prior to the PVD questionnaire, the procedure was identical to that of Experiment 1, we combined the data concerning the implicit and explicit attitudes of Tokyo residents gathered in Experiments 1 and 2. The correlation between the *D* scores and the relative likeability ratings for products from Fukushima was weakly negative (*n* = 80, *B_10_* = 1/1.45, *r* = −0.20), although the evidence anecdotally favored the null hypothesis.

### Discussion

First, the implicit attitudes toward agricultural and aquatic products from Fukushima were again negative relative to those from Saga; internal consistency was now acceptable. Second, the explicit attitudes toward products from Fukushima were consistently positive and hence dissociated from the implicit attitudes. Thus, the findings of Experiment 1 were robustly supported by those of Experiment 2.

Third, as predicted, we found a negative correlation between the *D* score and germ aversion, but it was not statistically supported. We consider that the absence of the statistical support is likely to be due to the extreme *D* score; that is, the correlation was undetectable due to the ceiling effect. After dichotomizing the data *via* a median split, we identified moderately negative implicit attitudes toward Fukushima among participants with low aversion to germs compared to participants with high aversion to germs. Furthermore, although this effect was moderate among participants with low aversion to germs, we detected a large effect in the participants with high aversion to germs, based on Cohen (1969). Although the median split is controversial (see Rucker et al., 2015), we would like to emphasize that it demonstrated a consistent trend with the correlation. On the other hand, the negative implicit attitudes toward Fukushima, including the effect size, were independent of the perceived infectability. These results can be interpreted in the context of the behavioral immune system (Kurzban and Leary, 2001; Schaller and Duncan, 2007; Duncan et al., 2009; Schaller and Park, 2011). As the perceived infectability in PVD reflects the self-perceived susceptibility to infection, it might be less relevant to radioactive contamination, which is not contagious. However, the aversion to germs in PVD reflects emotional discomfort in the presence of potential disease transmission vectors. Thus, converging evidence suggests that the participants of this study would have aversive affective responses to products from Fukushima so that they can avoid the invisible threat of radiation.

In terms of the correlation between implicit and explicit attitudes, there was a negative trend among the participants residing in Tokyo, although this was not supported by any statistical evidence. Therefore, current evidence suggests that the implicit attitudes were dissociated from, but not opposed to, the explicit attitudes; more work is needed with respect to this issue.

## General Discussion

We investigated the following three questions: (1) Do consumers have negative implicit attitudes toward agricultural and aquatic products from Fukushima? (2) Are these attitudes independent of their explicit attitudes? (3) Are they modulated by residential area? The answers to these questions are all yes. Although Japanese participants consistently had positive explicit attitudes toward products from Fukushima, relative to those from Saga, their implicit attitudes toward Fukushima were reliably negative. These were more predominant in the region near Fukushima (i.e., Tokyo) than in the region further away (i.e., Hiroshima). The results of recent research and market surveys (Miura et al., 2016; Kudo and Nagaya, 2017; Ministry of Agriculture, Forestry, and Fisheries, 2018) suggest that the current consumption situation embraces the apparent contradiction that products from Fukushima are somehow avoided by consumers who have no negative attitudes toward them. Focusing on the implicit attitudes and where consumers live, we succeeded in demonstrating a stepping stone toward the resolution of this paradox. Implicit attitudes may be associated with hesitancy to purchase products from Fukushima.

Furthermore, we explored individual differences in the formation of these implicit attitudes toward products from Fukushima in terms of the PVD (Duncan et al., 2009). The results of our analyses suggest that the negative implicit attitudes toward Fukushima products were attenuated (but still persistent) in participants with relatively low germ aversion in PVD. However, there was a large effect size among participants with high aversion to germs. Previous research suggests that we establish negative implicit attitudes toward foreigners or outside groups based on threat-connoting cues (Faulkner et al., 2004; Duncan et al., 2009; Huang et al., 2011). In this study, the label “Fukushima products” might serve as a threat-connoting cue, which then activates the behavioral immune system. This is our evolutionary adaptive disease-avoidance mechanism (Kurzban and Leary, 2001; Schaller and Duncan, 2007; Schaller, 2011; Schaller and Park, 2011). Because radiation is a serious threat to our health and survival that is also invisible, like a pathogen, it is plausible that consumers promote aversive, cautious (sometimes overcautious) attitudes toward products from Fukushima to minimize the impact of errors in judgment (they avoid making a type II false-negative error in terms of error management theory, Haselton and Buss, 2000). As a result, consumers’ attitudes are generally biased toward type I false-positive errors. This response is caused by the behavioral immune system (Oaten et al., 2009; Schaller, 2011; Kouznetsova et al., 2012; Miller and Maner, 2012). It has been suggested that the aversive, cautious response varies not only between individuals but also across external contexts, such as geographical region (Schaller and Murray, 2008). Our current findings (i.e., that negative implicit attitudes reliably observed in the region near Fukushima compared to the region far away) are consistent with this psychological survival function. Thus far, it has been shown that individuals’ anxiety and knowledge regarding radiation risk interactively influence their attitudes toward products from radioactive contamination areas (Miura et al., 2016). Developing this knowledge, the results of this study suggest that it is not just these factors but also individual differences in PVD that contribute to consumers’ attitudes.

It is noteworthy that implicit measures have been criticized for their proneness to measurement error, faking, or context dependency (see Gawronski and De Houwer, 2014; Gawronski and Hahn, 2019, for review). Although we used the conventional instrument in this study, it is important to investigate implicit attitudes with other instruments in future research, which would strengthen the current evidence.

Our results provide fundamental evidence supporting our hypothesis that it is implicit rather than explicit negative attitudes that elicit hesitancy about purchasing products from Fukushima. Future research is needed to validate our hypothesis more thoroughly. We will investigate whether the findings presented in this paper do indeed underpin the hesitation toward products from Fukushima. In fact, PVD has previously been reported to relate to consumers’ intentions to buy secondhand products (Kapitan and Bhargave, 2013). This supports our hypothesis. Future studies should determine whether a more negative implicit attitude toward Fukushima products is associated with a greater hesitancy to purchase these products.

## Author Contributions

OT and AA contributed conception and design of the study; OT, JQ, and DT conducted the experiments. OT, DT, and AA performed the statistical analysis. OT wrote the first draft of the manuscript. All authors read and approved the submitted version.

### Conflict of Interest Statement

The authors declare that the research was conducted in the absence of any commercial or financial relationships that could be construed as a potential conflict of interest.
